# Anti-obesity and lipid-lowering effects of mountain caviar extract and its principal saponin momordin Ic via delayed gastric emptying and GLP-1 secretion independent of energy expenditure

**DOI:** 10.1007/s11418-026-02052-3

**Published:** 2026-06-16

**Authors:** Ryuya Takada, Shogo Takeda, Hiroshi Shimoda, Motomitsu Tsukumo, Kazutaka Kubota, Hisashi Matsuda, Toshio Morikawa

**Affiliations:** 1 Pharmaceutical Research and Technology Institute, Kindai University, 3-4-1 Kowakae, Higashiōsaka, Osaka 577-8502 Japan; 2 Antiaging Center, Kindai University, 3-4-1 Kowakae, Higashiōsaka, Osaka 577-8502 Japan; 3 Oryza Oil & Fat Chemical Co., Ltd., 1 Numata, Kitagata-cho, Ichinomiya, Aichi 493-8001 Japan; 4 ES Tech Kyoto, 15 Shimogamo Morimoto-cho, Sakyo-ku, Kyoto, 606-0805 Japan; 5 Kyoto Organic Chemical Lab., 15 Shimogamo Morimoto-cho, Sakyo-ku, Kyoto, 606-0805 Japan; 6 Research Institute for Production Development, 15 Shimogamo Morimoto-cho, Sakyo-ku, Kyoto, 606-0805 Japan

**Keywords:** Mountain caviar, Anti-hyperlipidemic activity, Momordin Ic, Cholesterol metabolism, Doubly labeled water method, Total energy expenditure

## Abstract

The fruit of *Bassia scoparia* (L.) A. J. Scott [syn*. Kochia scoparia* (L.) Schrad.], commonly known as mountain caviar, are traditionally consumed as both food and medicine. Oral administration of 65% aqueous ethanol mountain caviar extract (MCE, 125–250 mg/kg) significantly suppressed postprandial plasma triglyceride (TG) elevation in olive oil-loaded mice. Among isolated saponins, the principal saponin momordin Ic exhibited significant antihyperlipidemic effect at 20 mg/kg. HPLC quantitative analysis using charged aerosol detector revealed that momordin Ic accounted for 12.6% of the MCE. Mechanistic studies demonstrated that momordin Ic markedly delayed gastric emptying (GE) without inhibiting pancreatic lipase activity. Moreover, momordin Ic significantly enhanced plasma glucagon-like peptide-1 (GLP-1) levels. In a high-fat diet-fed (HFD) mice model, 14-day administration of MCE suppressed body weight gain. Similarly, momordin Ic (20 mg/kg/day) significantly reduced visceral fat accumulation, liver cholesterol content, and plasma low-density lipoprotein/very low-density lipoprotein (LDL/VLDL) cholesterol levels, with a tendency to decrease plasma TG and total cholesterol. Notably, total energy expenditure measured using the doubly labeled water method was not significantly altered by MCE. These findings indicate that MCE and momordin Ic exert anti-obesity and lipid-lowering effects primarily through the suppression of dietary fat absorption and partly delayed GE, rather than by enhancing energy expenditure.

**Graphical abstract:**

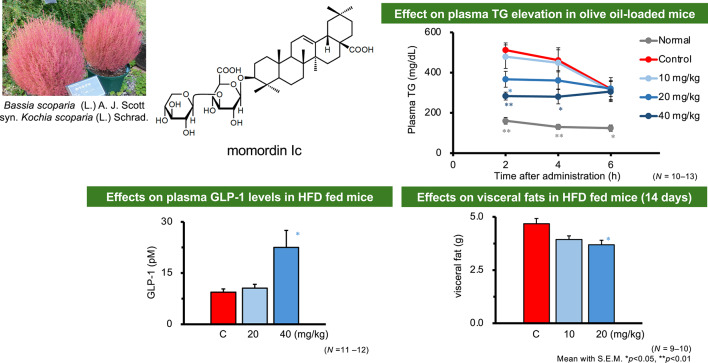

**Supplementary Information:**

The online version contains supplementary material available at 10.1007/s11418-026-02052-3.

## Introduction

Kochia [*Bassia scoparia* (L.) A. J. Scott, syn. *Kochia scoparia* (L.) Schrad.] is a large annual potherb in the family Amaranthaceae (Chenopodiaceae) widely distributed in Europe, Asia, naturalized Africa, Australia, and North and South America [1–3]. Because of its adorable appearance, it is known as “*Houkigi*” in Japan and is cultivated as an ornamental plant, whereas it is the most invasive agricultural weeds in Canada and the United States [4, 5]. The fruit of this plant, Kochiae Fructus, is listed as top-grade medicinal material of the oldest Chinese medicinal book “Shen-Nung’s Herbal Classic of Materia Medica” and has been used as a tonic, diuretic, analgesic, and antidote and for the treatment of cutaneous pruritus in traditional Chinese and Japanese medicinal preparations [2, 3, 6–10]. As an edible ingredient, Kochiae Fructus is also known as mountain caviar. In Akita Prefecture, a traditional local cuisine ingredient “Tonburi” is made from mountain caviar, named for its close resemblance to caviar in both physical appearance and texture [6–8, 11]. The plant exhibits range of pharmacological activities, including anti-inflammatory [2, 3, 10, 12–15], anti-pruritogenic [16, 17], antinociceptive [12], anti-allergic [18], hypoglycemic [2, 3, 7, 10, 19], anti-obese [2, 8], anticancer [2, 3, 10, 20–24], antibacterial [2, 25], antifungal [3, 10, 26, 27], antioxidant [2, 10, 28, 29], and hepatoprotective [10, 30] activities. Our previous studies on mountain caviars showed that it suppresses blood glucose elevation in glucose tolerance tests in rats [7, 11]. Its mechanism of action may involve inhibition of glucose absorption by the principal saponin constituent momordin Ic (**1**) [7, 11, 31–33], as well as suppression of gastric emptying (GE) [11, 33–35], acceleration of gastrointestinal transit (GIT) [33, 36, 37], and sodium-dependent glucose transporters in the intestinal mucosa [11]. In a clinical oral glucose tolerance test, participants receiving two capsules containing mountain caviar extract (MCE; 25 mg/capsule) showed a significant reduction in the maximum blood glucose concentration (*C*_max_), the predefined primary outcome [19]. In this study, we examined the effects of MCE and **1** on lipid absorption in olive oil- and high-fat diet (HFD)-fed mouse models. Furthermore, the effects of MCE and **1** on total energy expenditure (TEE) of mice were examined using the doubly labeled water (DLW) method.

## Results and discussion

### Preparation of MCE and quantitative analysis of momordin Ic (1)

The dried mountain caviar was finely crushed and extracted using 65% w/w aqueous ethanol at 70 °C for 2 h. The extracted solvent was evaporated at 50 °C under reduced pressure to obtain MCE (11.33%), as described previously [11]. Typical HPLC chromatograms of the standard solution of the principal constituent momordin Ic (**1**), which was isolated from the methanol extract of mountain caviar (vide infra), and a sample solution of MCE generated using a charged aerosol detector (CAD) were shown in Figure S1. A peak was observed at *t*_R_ 10.98 min and was unambiguously assigned by comparison of its retention time with that of authentic specimen [6, 7, 11]. According to the established protocol, the content of **1** in MCE was 12.6%.

### Effects of MCE on plasma triglyceride (TG) elevation in olive oil-loaded mice

We previously reported that several naturally occurring products such as sesquiterpene glycosides obtained from artichoke (leaves of *Cynara scolymus* L.) [38], diterpenes from sage (leaves of *Salvia officinalis* L.) [39], saponins from tea flower (flower buds of *Camellia sinensis* (L.) Kuntze) [33, 40, 41], daisy flower (flowers of *Bellis perennis* L.) [43, 44], pericarps of *Sapindus rarak* DC. [45], and maté (leaves of *Ilex paraguariensis* A. St. Hil.) [46], and oligostilbenes from bark of *Shorea roxburghii* G. Don [47] suppressed plasma TG elevation in olive oil-loaded mice. Similarly, we found that MCE significantly suppressed plasma TG elevation in mice 2 h after the administration of olive oil at a dose of 125 mg/kg, *p.o.*, as shown in Table 1.

**Table 1:** Inhibitory effects of MCE, momordin Ic (**1**) and related compounds (**2**, **1a**) on plasma TG elevation in olive oil-loaded mice

Treatment	Dose (mg/kg, *p.o.*)	*N*	Plasma triglyceride (mg/dL)			AUC (mg h/dL)
			2.0 h	4.0 h	6.0 h	
Normal	–	5	217.6 ± 18.5**	183.6 ± 9.2**	165.9 ± 10.9	750.8 ± 42.2**
Control	–	6	673.1 ± 31.0	639.7 ± 39.9	335.6 ± 40.5	2288.0 ± 141.5
MCE	125	7	392.6 ± 49.4**	506.0 ± 65.8	573.9 ± 52.9**	1978.4 ± 204.6
	250	7	320.0 ± 40.1**	455.8 ± 62.7*	534.2 ± 70.0*	1765.8 ± 184.9
	500	7	347.5 ± 46.9**	308.4 ± 18.4**	265.1 ± 9.1	1229.5 ± 74.5**
Normal	–	10	160.9 ± 16.5**	130.5 ± 13.0**	124.3 ± 15.4*	546.2 ± 52.6**
Control	–	11	512.1 ± 35.8	461.7 ± 51.9	318.7 ± 36.6	1754.1 ± 154.3
Momordin Ic (**1**)	10	13	479.3 ± 58.4	449.0 ± 74.7	310.9 ± 50.1	1688.2 ± 237.6
	20	12	367.0 ± 39.5*	361.3 ± 42.7	320.6 ± 54.5	1410.0 ± 100.5
	40	13	283.7 ± 19.7**	280.5 ± 35.6*	306.0 ± 50.3	1150.6 ± 126.8*
Normal	–	5	161.0 ± 8.4**	125.0 ± 4.0**	112.3 ± 8.7**	523.4 ± 17.1**
Control	–	7	537.0 ± 50.8	519.2 ± 52.4	291.1 ± 33.7	1866.6 ± 157.5
Momordin IIc (**2**)	20	6	551.6 ± 27.7	524.3 ± 46.0	297.9 ± 52.7	1858.2 ± 165.5
	40	7	480.4 ± 51.5	528.0 ± 53.7	276.8 ± 16.5	1813.1 ± 159.9
Normal	–	4	232.6 ± 17.5**	155.0 ± 13.8**	156.9 ± 12.2	699.5 ± 55.5**
Control	–	5	556.1 ± 50.4	551.6 ± 71.9	348.1 ± 35.3	2000.7 ± 209.3
Oleanolic acid (**1a**)	10	4	521.9 ± 60.4	573.4 ± 75.8	286.9 ± 66.2	1955.6 ± 241.5
	20	5	549.2 ± 57.4	538.2 ± 54.6	336.1 ± 12.2	1961.7 ± 151.7
	40	5	552.0 ± 46.7	426.7 ± 85.7	355.8 ± 104.1	1761.2 ± 308.0
Normal	–	6	212.8 ± 11.7**	160.7 ± 9.3**	135.8 ± 8.7**	670.0 ± 29.5**
Control	–	7	560.3 ± 55.6	524.9 ± 57.1	357.1 ± 75.9	1967.3 ± 226.8
Orlistat	5	8	562.3 ± 39.1	505.4 ± 72.8	215.1 ± 36.1*	1788.2 ± 173.6
	10	9	366.9 ± 53.2**	342.2 ± 45.8*	166.5 ± 12.6**	1217.8 ± 115.4**
	20	10	236.4 ± 18.4**	167.0 ± 12.7**	153.0 ± 9.5**	722.4 ± 49.0**

Each value represents the mean ± SEM

Significantly different from the control group, *p < 0.05, **p < 0.01

### Effects of momordin Ic (1) and related compounds on plasma TG levels in olive oil-loaded mice

**Fig. 1:**
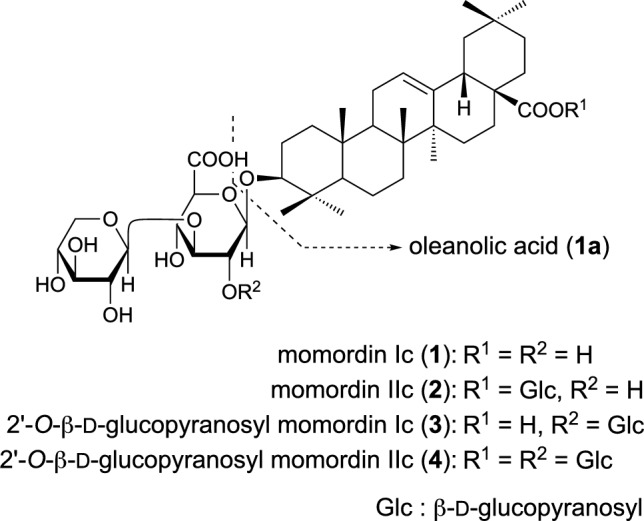
Structures of momordin Ic (**1**) and related compounds (**2**–**4**, **1a**)

Dried mountain caviar was extracted with methanol (MeOH) under reflux to yield a methanol extract (14.47% from the dried plant material). The extract was subjected to normal-phase silica gel chromatography, reversed-phase octadecylsilyl (ODS)-column chromatography, and HPLC to give four oleanane-type triterpene saponin constituents, momordin Ic (**1**, 0.74%) [4, 5, 47, 48], momordin IIc (**2**, 0.11%) [4, 5, 47, 48], 2′-*O*-β-d-glucopyranosyl momordin Ic (**3**, 0.021%) [2, 6, 49], and 2′-*O*-β-d-glucopyranosyl momordin IIc (**4**, 0.023%) [2, 6, 49] (Fig. 1).

As shown in Table 1, momordin Ic (**1**) significantly suppressed plasma TG elevation 2 h after olive oil administration at a dose of 20 mg/kg, *p.o*. However, momodin IIc (**2**), 28-*O*-β-d-glucopyranosyl ester of **1**, and the common aglycone oleanolic acid (**1a**) showed no significant effect up to the dose of 40 mg/kg.

### Mechanisms of action of inhibitory effects on TG absorption

Prolonged retention of lipids in the stomach and delayed GE are proposed to suppress the intestinal TG absorption [33–35, 38, 39, 50, 51]. Pancreatic lipase, which hydrolyzes TG to monoglycerides and free fatty acids (FFA) prior to absorption in the small intestine, plays an important role in lipid digestion. Therefore, inhibition of pancreatic lipase activity is expected to reduce TG absorption [52, 53]. Suppression of GE together with pancreatic lipase inhibition helps regulate postprandial plasma TG elevation and may aid in the prevention or amelioration of dyslipidemia. As shown in Table 2, momordin Ic (**1**) significantly suppressed GE in mice 2 h after administration of olive oil at a dose of 40 mg/kg, *p.o*. In addition, **1** moderately inhibited enzymatic pancreatic lipase activity (IC_50_ = 256.1 µM, Table S1).

**Table 2:** Effects of Momordin Ic (**1**) on GE in olive oil-loaded mice

Treatment	Dose (mg/kg, *p.o.*)	*N*	Gastric emptying (%)
Control	–	6	77.3 ± 1.3
Momordin Ic (**1**)	20	5	67.9 ± 4.3
	40	5	28.0 ± 9.9**
Control	–	11	79.7 ± 1.4
Butyl scopolamine	20	9	80.1 ± 0.8
	50	5	71.6 ± 3.2
	100	5	57.2 ± 7.7*
Atropine	5	8	58.9 ± 6.0*
	10	8	52.9 ± 9.0**

Each value represents the mean ± SEM

Significantly different from the control group, *p < 0.05, **p < 0.01

Han *et al.* reported that the anti-obesity effects of *K. scoparia* extract are partly mediated by delayed intestinal absorption of dietary fat through the inhibition of pancreatic lipase activity by its saponins such as momordin Ic (**1**) [8]. In contrast our findings, suggest that pancreatic lipase inhibitory activity was limited and partly depended on the inhibition of GE by **1** as a mechanism of action. In addition, **1** at a dose of 25 mg/kg *p.o*. has been reported to accelerate GIT in carboxymethyl cellulose treated mice [36, 37]. Therefore, we examined the effect of **1** on GIT in an olive oil-loaded mice model, however, observed no significant acceleration on activity (Table S2).

Glucagon-like peptide 1 (GLP-1) and cholecystokinin (CCK) secreted from intestinal I-cells and L-cells stimulate their respective receptors. This secretion is mediated through the afferent vagal nerves and nucleus tractus solitarius to reduce the expression of neuropeptide Y and aguti-related peptide, ultimately suppressing appetite. Stimulation of the 5-hydroxytryptamine 2B (5-HT_2B_) receptor in the stomach via 5-HT released from intestinal chromaffin cells inhibits the release of ghrelin, which stimulates appetite through the afferent vagal nerves, whereas stimulation of the 5HT_2C_ receptor in the hypothalamus stimulates proopiomelanocortin neurons to reduce appetite [54–59]. We therefore examined effects of MCE and **1** on GLP-1 and CCK secretions. As shown in Table 3, MCE (500 mg/kg, *p.o*.) tended to increase GLP-1 levels but not affected to CCK levels. Furthermore, **1** (40 mg/kg, *p.o.*) significantly increased plasma GLP-1 levels. Consequently, **1** inhibited plasma TG levels after olive oil loading in mice without inhibiting pancreatic lipase. GLP-1 and CCK inhibit GE [60, 61], suggesting that enhancement of GLP-1 release also contributes to GE inhibition by **1**.

**Table 3:** Effects of MCE and momordin Ic (**1**) on plasma GLP-1 and CCK levels in mice

Treatment	Dose (mg/kg, *p.o.*)	*N*	Food intake (g/mouse)^a^	Plasma GLP-1 (pM)	Plasma CCK (ng/mL)
Control	–	11	0.89	9.4 ± 0.9	2.23 ± 0.25
MCE	250	12	0.44	13.5 ± 2.3	2.28 ± 0.28
	500	11	0.26	19.5 ± 3.7	2.36 ± 0.30
Momordin Ic (**1**)	20	12	0.65	10.6 ± 1.1	2.37 ± 0.31
	40	11	0.40	22.5 ± 6.7*	2.35 ± 0.28

Each value represents the mean ± SEM

Significantly different from the control group, *p < 0.05

^a^Each value was calculated based on the food intake (g) measured from the two groups housed in two cages

### Anti-obesity effects in high-fat diet (HFD)-fed mice

**Fig. 2:**
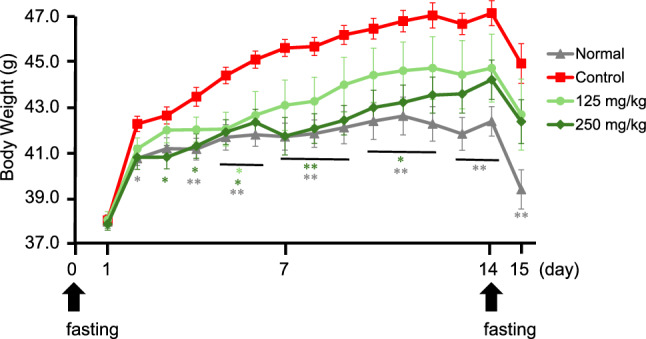
Effects of MCE on body weight gain in high-fat diet-fed mice. Male ddY mice were fed a high-fat diet (HFD-60) or normal diet (AIN-93M) for 14 days. Each value represents the mean ± SEM (*N* = 8). Significant difference where *p < 0.05 or **p < 0.01 was compared with controls

Next, we examined the effects of MCE on body weight gain in HFD-fed mice. As shown in Fig. 2, MCE (250 mg/kg/day, *p.o*.) inhibited body weight gain 3–12 days after administration. In addition, this extract tended to suppress visceral fat weight (total weight of perirenal, epididymal, and mesenteric fat) without any obvious toxic effects, as shown in Table 4.

**Table 4:** Effects of MCE on visceral fat, liver weight and plasma and liver biochemicals in HFD fed mice

Treatment	Dose (mg/kg/day, *p.o.*)	*N*	Visceral fat^a^ (g)	Perirenal fat (g)	Epididymal fat (g)	Mesenteric fat (g)	Liver weight (g)
Normal	–	8	1.63 ± 0.18**	0.19 ± 0.04**	0.95 ± 0.20**	0.60 ± 0.09	1.39 ± 0.03
Control	–	8	3.42 ± 0.31	0.54 ± 0.07	1.91 ± 0.18	0.98 ± 0.12	1.57 ± 0.05
MCE	125	8	2.87 ± 0.35	0.37 ± 0.05	1.66 ± 0.20	0.85 ± 0.15	1.66 ± 0.10
	250	8	2.99 ± 0.40	0.42 ± 0.05	1.75 ± 0.24	0.82 ± 0.15	1.44 ± 0.06

Plasma							
TG (mg/dL)	CHO (mg/dL)	FFA (mg/dL)	Glc (mg/dL)	AST (mg/dL)	ALT (mg/dL)	HDL (mg/dL)	LDL/VLDL (mg/dL)
233.1 ± 37.7**	169.7 ± 17.3	1.57 ± 0.08*	110.1 ± 16.4**	60.6 ± 12.3	15.5 ± 3.3*	82.3 ± 4.6*	23.8 ± 1.6
112.6 ± 12.3	207.0 ± 44.9	1.07 ± 0.13	171.8 ± 12.5	95.5 ± 28.5	39.6 ± 18.0	120.0 ± 16.2	27.0 ± 7.2
145.2 ± 18.0	149.8 ± 13.5	0.91 ± 0.09	173.5 ± 17.1	43.2 ± 5.1*	11.8 ± 1.0*	74.9 ± 8.1**	31.2 ± 7.2
126.7 ± 15.3	198.9 ± 8.0	0.93 ± 0.06	179.2 ± 14.1	63.1 ± 13.7	16.1 ± 1.9	93.7 ± 4.5	25.1 ± 0.8

Liver		
Fat (mg/g)	TG (mg/g)	CHO (mg/g)
216.0 ± 15.9	20.4 ± 2.6*	1.08 ± 0.05*
246.9 ± 15.6	40.6 ± 5.0	1.33 ± 0.06
202.5 ± 12.6	25.4 ± 4.7	1.42 ± 0.07
191.2 ± 11.8*	32.5 ± 7.5	1.60 ± 0.07*

Each value represents the mean ± SEM

Significantly different from the control group, *p < 0.05, **p < 0.01

^a^The weight of visceral fat was estimated as the total weight of perirenal, epididymal, and mesenteric fats

**Fig. 3:**
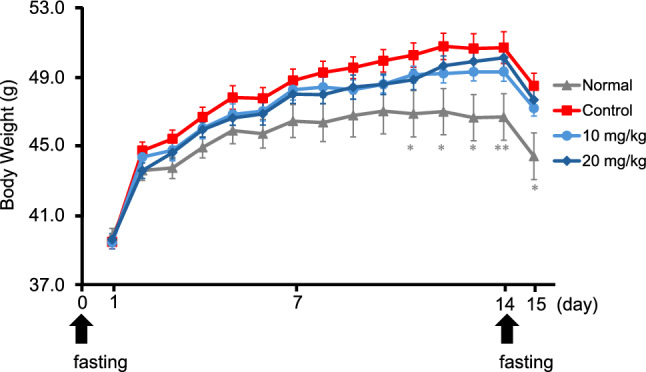
Effects of momordin Ic (**1**) on body weight gain in high-fat diet-fed mice. Male ddY mice were fed a high-fat diet (HFD-60) or normal diet (AIN-93M) for 14 days. Each value represents the mean ± SEM (*N* = 9–10). Significant difference where *p < 0.05 or **p < 0.01 was compared with controls

As shown in Fig. 3 and Table 5, 14-day continuous administration of momordin Ic (**1**, 20 mg/kg/day) significantly inhibited visceral fat, plasma low-density lipoprotein/very low-density lipoprotein (LDL/VLDL) cholesterol (CHO), and liver fat levels. However, in contrast to the acute experiment conducted under fasted conditions, no suppressive effect of **1** on food intake was observed in this experiment (Table S3). This difference may be attributable to the difference between fasted conditions in the acute experiment and non-fasted conditions in the HFD-fed experiment. In this experiment, compound **1** reduced the accumulation of visceral fat and is suggested to ameliorate abnormalities in carbohydrate metabolism induced by HFD.

**Table 5:** Effects of momordin Ic (**1**) on visceral fat, liver weight and plasma and liver biochemicals in HFD fed mice

Treatment	Dose (mg/kg/day, *p.o.*)	*N*	Visceral fat^a^ (g)	Perirenal fat (g)	Epididymal fat (g)	Mesenteric fat (g)	Liver weight (g)
Normal	–	9	2.68 ± 0.35**	0.48 ± 0.05**	1.52 ± 0.22**	1.02 ± 0.11**	1.51 ± 0.07
Control	–	9	4.68 ± 0.24	0.71 ± 0.04	2.49 ± 0.14	1.48 ± 0.13	1.60 ± 0.03
momordin Ic (**1**)	10	10	3.93 ± 0.18	0.64 ± 0.04	2.26 ± 0.11	1.02 ± 0.07**	1.50 ± 0.04
	20	10	3.68 ± 0.22*	0.58 ± 0.03**	2.04 ±0.13	1.06 ±0.09*	1.53 ± 0.06

Plasma							
TG (mg/dL)	CHO (mg/dL)	FFA (mg/dL)	Glc (mg/dL)	AST (mg/dL)	ALT (mg/dL)	HDL (mg/dL)	LDL/VLDL (mg/dL)
169.5 ± 18.8*	212.3 ± 26.6	1.34 ± 0.06**	161.8 ± 21.2	80.7 ± 17.1	35.0 ± 6.4	112.1 ± 9.6	41.5 ± 4.4**
117.0 ± 13.6	262.7 ± 10.9	1.00 ± 0.09	160.9 ± 11.8	53.0 ± 2.7	27.9 ± 2.7	115.5 ± 7.6	61.9 ± 4.8
120.7 ± 15.2	237.5 ± 21.7	1.09 ± 0.07	176.1 ± 12.1	91.5 ± 16.7	28.5 ± 2.7	131.1 ± 9.3	43.1 ± 3.2**
94.5 ± 6.2	207.3 ± 17.9	0.88 ± 0.05	165.3 ± 12.7	90.1 ± 14.1	41.3 ± 5.9	112.5 ± 9.4	42.2 ± 3.7**

Liver		
Fat (mg/g)	TG (mg/g)	CHO (mg/g)
165.0 ± 12.1*	40.0 ± 2.4**	1.41 ± 0.12
236.7 ± 23.1	47.4 ± 0.4	1.79 ± 0.11
194.4 ± 13.3	46.8 ± 1.1	2.32 ± 0.11
190.8 ± 17.3	47.1 ± 0.9	2.42 ± 0.23*

Each value represents the mean ± SEM

Significantly different from the control group, *p < 0.05, **p < 0.01

^a^The weight of visceral fat was estimated as the total weight of perirenal, epididymal, and mesenteric fats

### Effect of MCE on total energy expenditure (TEE) in HFD-fed mice

Recently, the DLW method was used as a standard method for simplified measurement of TEE in human clinical studies [62]. In this study, the DLW method was applied in animal experiments to evaluate energy metabolism [63–68]. As shown in Table 6, MCE (125 and 250 mg/kg/day) did not significantly increase TEE. In addition, momordin Ic (**1**, 10 and 20 mg/kg/day) did not increase the TEE, although the pre-administration period of **1** before DLW administration was 3 days, rather than 11 days (Table S4). Further examination of **1** under the same experimental conditions is required.

**Table 6:** Effects of MCE on TEE using DLW in HFD fed mice

Treatment	Dose (mg/kg/day, *p.o.*)	N	Body weight (g)	TBW (mol)	k_d_ (× 10^–3^/h)	k_o_ (× 10^–3^/h)	rCO_2_ (mol/day)	TEE (kcal/day)	TEE (kcal/kg/day)
Normal	–	5	41.9 ± 2.7	1.58 ± 0.10	7.9 ± 1.0	15.1 ± 1.2	0.123 ± 0.013	15.8 ± 1.6	382.8 ± 46.0
Control	–	6	47.1 ± 2.1	1.61 ± 0.08	8.8 ± 0.6	15.1 ± 0.8	0.117 ± 0.004	16.7 ± 0.5	358.0 ± 19.1
MCE	125	5	47.3 ± 2.3	1.75 ± 0.08	6.9 ± 0.7	12.8 ± 1.0	0.120 ± 0.003	17.1 ± 0.4	364.1 ± 20.6
	250	6	42.5 ± 2.2	1.56 ± 0.10	8.5 ± 1.2	14.5 ± 1.5	0.107 ± 0.006	15.3 ± 0.9	365.1 ± 30.2

Each value represents the mean ± SEM. Calculation procedure was described in Supplementary Information

*TBW* total body water (mol), *k*_*d*_ rate constant of ^2^H (× 10^–3^/h), *k*_*o*_ rate constant of ^18^O (× 10^–3^/h), *rCO*_*2*_ CO_2_ production (mol/day)

## Conclusion

This study demonstrates that MCE and its principal saponin, momordin Ic (**1**), exert significant lipid-lowering and anti-obesity effects in murine models. Both MCE and **1** suppressed postprandial plasma TG elevation following olive oil loading, partly through delayed GE rather than by pancreatic lipase inhibition. Furthermore, **1** enhanced GLP-1 secretion, suggesting the involvement of gut-derived hormonal signaling in appetite regulation. In HFD-fed mice, continuous administration of MCE attenuated body weight gain, whereas **1** significantly reduced visceral fat accumulation, liver fat and CHO, and plasma LDL/VLDL CHO levels. Importantly, these metabolic improvements occurred without a concomitant increase in TEE, as assessed using the DLW method. Collectively, these results indicate that mountain caviar and **1** ameliorated diet-induced obesity and dyslipidemia by modulating gastrointestinal function and satiety-related hormonal pathways, independent on energy consumption. Further studies are warranted to determine the detailed molecular mechanisms underlying GLP-1-mediated signaling and assess their clinical significance.

## Materials and methods

### General

The following instruments were used to obtain spectroscopic data: ^1^H NMR spectra, Ascend Evo 800 (800 MHz, Bruker Corporation, Billerica, MA, USA), JNM-ECA800 (800 MHz), and JNM-ECS400 and JNM-AL400 (400 MHz) (JEOL Ltd., Tokyo, Japan) spectrometers; ^13^C NMR spectra, Ascend Evo 800 (200 MHz), JNM-ECA800 (200 MHz), and JNM-ECS400 and JNM-AL400 (100 MHz) spectrometers with tetramethylsilane (Tokyo Chemical Industry Co., Ltd., Tokyo, Japan) as an internal standard; ESIMS and HRESIMS, Exactive Plus mass spectrometer (Thermo Fisher Scientific, Waltham, MA, USA); HPLC detector, Shimadzu RID-6A refractive index (RI) detector, (Shimadzu Corporation, Kyoto, Japan); HPLC columns, Cosmosil 5C_18_-MS-II (Nacalai Tesque, Kyoto, Japan), 4.6 mm i.d. × 250 mm and 20 mm i.d. × 250 mm for analytical and preparative purposes, respectively, and CAPCELL PAK C18 (OSAKA SODA Co., Ltd., Osaka, Japan), 5 µm particle size, 4.6 mm i.d. × 250 mm for quantitative analysis purposes. The following experimental conditions were used for column chromatography (CC): normal-phase silica gel CC, silica gel 60N (Kanto Chemical Co., Ltd., Tokyo, Japan; 63–210 mesh, spherical, neutral); reversed-phase ODS CC, Chromatorex ODS DM1020T (Fuji Silysia Chemical, Ltd., Aichi, Japan; 100–200 mesh); TLC, pre-coated TLC plates with silica gel 60F_254_ (Merck, Darmstadt, Germany, 0.25 mm) (normal-phase) and silica gel RP-18 WF_254S_ (Merck, Darmstadt, Germany, 0.25 mm) (reversed-phase); reversed-phase HPTLC, pre-coated TLC plates with silica gel RP-18 WF_254S_ (Merck, Darmstadt, Germany, 0.25 mm). Detection was performed by spraying with 1% Ce(SO_4_)_2_–10% aqueous H_2_SO_4_ followed by heating.

### Reagents

Heparin, LabAssay™ Triglyceride, NEFA, Glucose, Cholesterol, AST and ALT, GLP-1 ELISA kit, and platinum catalyst (5% platinum on alumia) were purchased from FUJIFILM Wako Pure Chemical Co. (Osaka, Japan). Oil Red O was from Thermo Fisher Scientific (Waltham, MA, USA). Atropine, butyl scopolamine, triolein, 1,2-dipalmitoyl-*sn*-glycero-3-phosphocholine, orlistat, and linagliptin were from Tokyo Chemical Industry Co., Ltd. (Tokyo, Japan). Sodium taurocholate was from MP Biomedicals (Irvine, CA, USA). Porcine pancreatic lipase (L3126, type II) was from Sigma-Aldrich (St. Louis, MO, USA). HFD-60 and AIN-93M were from Oriental Yeast Co., Ltd. (Tokyo, Japan). CCK (26-33) (Non-Sulfated) (Human, Rat, Mouse, Canine)-EIA Kit (EK-069-04) was from Phoenix Pharmaceuticals Inc. (Burlingame, CA, USA). EnzyChrom™ HDL and LDL/VLDL Assay Kits (EHDL-100) were from BioAssay Systems (Hayward, CA, USA). H_2_^18^O (F03-0027, 99.5%) was from Taiyo Nippon Sanso Co. (Tokyo, Japan). ^2^H_2_O (7789-20-0, 99.9%) was from Cambridge Isotope Laboratories (Tewksbury, MA, USA).

### Plant material

The fruit of *B. scoparia* (syn. *K. scoparia*) cultivated in Xian, Shaanxi Province, China was identified by one of the authors (T.M.), as described in a previous report [11].

### Preparation of MCE

Dried mountain caviar was finely crushed and extracted with 65% w/w aqueous ethanol at 70 °C for 2 h. The extracted solvent was evaporated at 50 °C under reduced pressure to obtain MCE (11.33% from the dried material), as described previously [11].

### Quantitative determination of momordin Ic (1) in the MCE

#### Standard preparation

An accurately weighed 5.0 mg of momordin Ic (**1**) was introduced into a 10 mL volumetric flask, and methanol was added to make up the volume of the stock standard solution (500 µg/mL). The solution was filtered through a syringe filter (0.45 µm) and aliquots of 2.5 and 5.0 mL of the stock standard solution were transferred into 10 mL volumetric flask; the volume was made up with methanol for use as working solutions (125, 250, and 500 µg/mL, respectively) for constructing calibration curves. For calibration, an aliquot of 10 mL of each solution was injected into the HPLC system (*t*_R_ 10.98 min, Figure S1).

#### Sample preparation

An accurately weighed pulverized MCE and the methanol extract of mountain caviar (each approximately 40 mg, conversion with loss on drying) were introduced into a 20 mL volumetric flask, 15 mL of methanol was added, and ultrasonic treatment was performed for 30 min. Methanol was added to make up the final concentration of the stock solution to (2 mg/mL). The solution was filtered through a syringe filter (0.45 µm), and a 10 µL aliquot was injected to HPLC analysis.

### HPLC instruments and conditions

All analytical experiments were performed using an LC-20A series Prominence HPLC system (Shimadzu), which consisted of a charged aerosol detector (CAD, Corona Veo, Thermo Fisher Scientific), binary pump, degasser, autosampler, thermostated column compartment, and control module. Chromatographic separation was performed on a CAPCELL PAK C18 (5 µm particle size, 4.6 mm i.d. × 250 mm, OSAKA SODA Co., Ltd.) operated at 35 °C with mobile phase MeOH–0.2% aqueous AcOH (83:17, v/v). The flow rate was 1.0 mL/min and the injection volume was 10 mL (Figure S1).

### Calibration

Standard curves were prepared at three concentrations in the range 125–500 µg/mL. Standard curves were generated on each day of analysis. The linearity of each compound was plotted using a linear regression of the peak area versus concentration. The coefficient of correlation (*R*^2^ = 0.9997) was used to determine the linearity (*y* = 23525439*x* + 872428) (Figure S1).

### Extraction and isolation

Dried mountain caviar (500 g) was extracted three times with methanol under reflux for 3 h. Evaporation of the combined extracts under reduced pressure yielded a MeOH extract (72.44 g, 14.5%). An aliquot (50.0 g) of the methanol extract was subjected to normal-phase silica gel CC [1.5 kg, CHCl_3_–MeOH–H_2_O (10:3:0.3 → 7:3:0.4 → 6:4:1, v/v/v) → EtOAc → MeOH] to produce six fractions [Fr. 1 (6.03 g), Fr. 2 (1.54 g), Fr. 3 (2.31 g), Fr. 4 (14.41 g), Fr. 5 (14.40 g), Fr. 6 (4.65 g)]. Fraction 4 (14.41 g) was subjected to reversed-phase silica gel CC [432 g, MeOH–H_2_O (60:40 → 70:30 → 90:10, v/v) → MeOH] to yield four fractions {Fr. 4-1 (6.39 g), Fr. 4-2 (260.0 mg), Fr. 4-3 [= momordin Ic (**1**, 3.71 g, 0.74%)] [47, 48] (Figures S2 and S3), and Fr. 4-4 (4.05 g)}. Fraction 5 (12.10 g) was subjected to reversed-phase silica gel CC [400 g, MeOH–H_2_O (60:40 → 70:30 → 80:20 → 90:10, v/v) → MeOH] to yield eight fractions {Fr. 5-1 (7.25 g), Fr. 5-2 (272.6 mg), Fr. 5-3 (115.0 mg), Fr. 5-4 [= 2′-*O*-β-d-glucopyranosyl momordin Ic (**3**,89.3 mg, 0.021%)] [49], Fr. 5-5 (147.8 mg), Fr. 5-6 (333.7 mg), Fr. 5-7 (86.7 mg), and Fr. 5-8 (78.6 mg)}. Fraction 5-2 (272.6 mg) was purified using HPLC [Cosmosil 5C_18_-MS-II, detection: RI, MeOH-1% aqueous AcOH (70:30, v/v)] to yield momordin IIc (**2**, 124.8 mg, 0.11%) [47, 48] and 2′-*O*-β-d-glucopyranosyl momordin IIc (**4**, 26.3 mg, 0.023%) [49]. These isolates were identified by comparing their physical and spectral data with those of authentic samples [6, 7].

### Animals

Male ddY mice were purchased from Kiwa Laboratory Animal Co., Ltd., (Wakayama, Japan). The animals were housed at a constant temperature of 23 ± 2 °C and fed a standard laboratory chow (EF, Oriental Yeast Co., Ltd., Tokyo, Japan). All experiments were performed using conscious mice, unless otherwise noted. The experimental protocol was approved by the Kindai University Committee for the Care and Use of Laboratory Animals (KAPR-2023-004, 2025-001, 2025-003).

### Effect on plasma TG elevation in olive oil-loaded mice

Each test sample was suspended in a 5% (w/v) acacia solution (10 mL/kg) and administered orally to fasted mice (6 weeks old, approximately 30 g), and olive oil (5 mL/kg) was administered *p.o.* for 30 min thereafter. Blood samples (approximately 0.3 mL) were collected in a polyethylene tube (1.5 mL) containing heparin (5 units/tube) from the infraorbital venous plexus at 2, 4, and 6 h after olive oil treatment. Plasma TG determined using a commercial kit (LabAssay™ Triglyceride) [33, 38–46]. The pancreatic lipase inhibitor orlistat was used as a reference compound.

### Effect on GE in olive oil-loaded mice

Experiments were performed as described in our previous reports [33–35, 38, 39, 50, 51] with slight modifications. Briefly, each test sample was suspended in a 5% (w/v) acacia solution (10 mL/kg) and administered orally to fasted mice (6 weeks old, approximately 30 g). Thirty minutes later, olive oil containing 0.05% Oil Red O as a marker was intragastrically administered (0.5 mL/kg) to conscious mice. After 2 h, the mice were euthanized by cervical dislocation under anesthesia. The abdominal cavity was opened and the gastroesophageal junction and pylorus were clamped, and the stomach was removed and the whole was homogenized with 10 mL of water. The homogenate was partitioned using 20 mL of EtOAc, and an aliquot of the EtOAc-soluble portion (1 mL) was dried with nitrogen to obtain a residue. The residue was dissolved in 2-propanol–DMSO (1:1, v/v, 200 µL), and the amount of Oil Red O was determined from the optical density (O.D.) at 518 nm using a microplate reader (SH-1000 Lab., Corona Electric Co., Ltd., Tokyo, Japan). The test sample was orally administrated 30 min prior to administering the olive oil solution. GE (%) during the 2-h period was calculated using the following equation:

$${\text{GE }}\left( \% \right) = 1 - \left( {\frac{{{\text{amount of Oil Red O in test sample}}}}{{{\text{amount of Oil Red O administered}}}}} \right) \times 100.$$

The anticholinergic agents, atropine and butyl scopolamine were used as reference compounds.

### Effect on pancreatic lipase activity

A suspension of triolein (80 mg), 1,2-dipalmitoyl-*sn*-glycero-3-phosphocholine (10 mg), and sodium taurocholate (5 mg) in 9 mL of 0.1 M Tris-HCl buffer (pH 7.0) containing 0.1 M NaCl was sonicated for 10 min. This sonicated substrate suspension (0.1 mL) in a test tube was pre-incubated with 5 µL of test sample in DMSO and 95 µL of Tris-HCl buffer for 3 min at 37 °C. An aliquot of porcine pancreatic lipase (L3126, type II, 250 µg/mL) or Tris-HCl buffer (50 µL) was added as a blank to start the reaction. After 30 min of incubation, the test tube was immediately immersed in boiling water for 2 min to stop the reaction, followed by cooling with iced water. FFA concentration was determined using a commercial kit (LabAssay™ NEFA) [39, 46, 52, 53]. Orlistat was used as a reference compound. IC_50_ was determined graphically.

### Effect on GIT in olive oil-loaded mice

GIT was evaluated as described in our previous reported [33, 36, 37, 41] with slight modifications. Briefly, each test sample was suspended in a 5% (w/v) acacia solution (10 mL/kg) and administered orally to fasted mice (6 weeks old, approximately 30 g). One hour later, olive oil containing 5% charcoal powder as a marker was intragastrically administered (0.5 mL/kg) to conscious mice. Each mouse group was euthanized by cervical dislocation under anesthesia at 30, 60, and 120 min. The abdominal cavity was opened, and the gastrointestinal tract was removed. The distance traveled by the marker was measured and expressed as a percentage of the total length of the small intestine from the pylorus to the caecum.

### Effects on plasma GLP-1 and CCK levels

Experiments were performed as described in our previous report [33, 41] with slight modifications. Male ddY mice (6 weeks old, approximately 30 g) were fed a HFD (HFD-60, 5.062 kcal/g) for seven days before the experiments. Each test sample was suspended in a 5% (w/v) acacia solution (10 mL/kg) and administered orally to fasted mice 45 min before the midnight satiety test. The mice were fed a HFD for 30 min, then a dipeptidyl peptidase-IV (DPP-IV) inhibitor linagliptin was administrated (10 mg/kg, *i.p.*). After 30 min, blood samples were collected from the infraorbital venous plexus, under anesthesia, and plasma GLP-1 and CCK levels were measured using ELISA kits.

### Measurements of body weight gain, liver and visceral fat weights, and plasma and liver biochemicals in the HFD-fed mice

The experiment was performed as previously reported with slight modifications [51]. Briefly, male ddY mice (10 weeks old) were fed either a normal diet (AIN-93M, 3.8 kcal/g) or a HFD (HFD-60) for 14 days. The test sample was suspended in a 5% acacia solution and administered orally using a metal orogastric tube once a day from 15:00–17:00. Body weight and food intake were measured daily, and the fecal TG content was measured weekly (Table S3). The mice were fasted for 20 h before days 1 and 15, and blood (approximately 0.2 mL) was collected from the infraorbital venous plexus under isoflurane anesthesia. Blood was immediately mixed with heparin sodium (10 units/tube). After centrifugation of the blood samples, plasma glucose, TG, FFA, total CHO, HDL CHO, and LDL/VLDL CHO levels were determined using commercial kits (LabAssay™ Triglyceride, Cholesterol, NEFA, Glucose, AST, and ALT and EnzyChrom™ HDL and LDL/VLDL Assay Kits). After 14 days, the mice were sacrificed by cervical dislocation under isoflurane anesthesia, and the visceral fat (perirenal, epididymal, and mesenteric fat) and the liver were removed and weighed. The crude fat, TG, and CHO contents in the liver were measured. Fecal samples were collected on days 4–7 and 11–14, weighed and TG levels were determined using a commercial kit (LabAssay™ Triglyceride).

### Measurement of TEE using DLW method

Male ddY mice (8–9 weeks old) were fed a normal diet (AIN-93M) or a HFD (HFD-60) for 14 days. The test sample was suspended in a 5% (w/v) acacia solution (10 mL/kg/day) and administered orally using a metal orogastric tube once per day from 15:00–17:00. The body weight and food intake were measured daily. DLW was administered 4 days before the end of the test. Blood samples were collected at 0, 4, 24, and 72 h after administration of DLW. After centrifugation of the blood samples, the plasma was diluted with water and measured Using Isotope Ratio Mass Spectrometry (IRMS, Hydra 20-22 with an automatic gas sampler, Sercon Ltd., Cheshire, UK). DLW was prepared as a mixture of H_2_^18^O (99.5%; Taiyo Nippon Sanso Co.), ^2^H_2_O (99.9%; Cambridge Isotope Laboratories), and water (0.75, 0.25, and 4 mL, respectively).

### Isotope analysis and calculations

The ^2^H/^1^H and ^18^O/^16^O isotope ratios in plasma samples were measured as described blew. Briefly, ^2^H enrichment was determined from the ^2^H/^1^H ratio of H_2_ generated by reducing test water samples (0.3 mL) in a evacuated flat bottom vial (Exetainer^®^ 12 mL vial, Labco Ltd., Lampeter, U.K.) with approximately 5 mg platinum catalyst (5% platinum on alumina, FUJIFILM Wako Pure Chemical Co.) in a insert tube (approximately 3.5 mm, i.d. × 30 mm, flat bottom) for 24 h, and ^18^O enrichment was calculated from the ^18^O/^16^O ratios of CO_2_ in the vial after equilibration with CO_2_ gas for 24 h. Stable isotopic standard waters [Natural Waters Standard (^2^H: − 51.84‰, ^18^O: − 7.46‰), Medium D Water Standard (+ 574.10‰), High D Water Standard (+ 1196.50‰), Medium ^18^O Water Standard (+ 100.32‰), High ^18^O Water Standard (+ 252.36‰), Taiyo Nippon Sanso Co., Tokyo, Japan] were used to evaluate each sample. The samples were measured in duplicate, and average values were used to calculate CO_2_ production (rCO_2_) and TEE [68] as described in the Supplementary Information.

### Statistical analysis

Values are expressed as the mean ± SEM. One-way analysis of variance followed by Dunnett’s test was used for statistical analyses. Probability (*p*) values less than 0.05 were considered significant.

## Supplementary Information

Below is the link to the electronic supplementary material.

Supplementary file1 (PDF 1019 KB)

## Abbreviations

MCE: 65% Aqueous ethanol mountain caviar extract
GE: Gastric emptying
GIT: Gastrointestinal transit
CAD: Charged aerosol detector
HFD: High-fat diet
5-HT: 5-Hydroxytryptamine
GLP-1: Glucagon-like peptide-1
CCK: Cholecystokinin
HDL: High-density lipoprotein
LDL/VLDL: Low-density lipoprotein/very low-density lipoprotein
CHO: Cholesterol
FFA: Free fatty acids
AST: Aspartate aminotransferase
ALT: Alanine aminotransferase
DLW: Doubly labeled water
TEE: Total energy expenditure
