# Supplementary material for: Anti-obesity and lipid-lowering effects of mountain caviar extract and its principal saponin momordin Ic via delayed gastric emptying and GLP-1 secretion independent of energy expenditure
Source: J Nat Med. 2026 Jun 16;80(4):1168–79. doi: 10.1007/s11418-026-02052-3 (PMC13350199; doi:10.1007/s11418-026-02052-3)
Supplement: Supplementary file 1 — Supplementary file1 (PDF 1019 KB) [file 11418_2026_2052_MOESM1_ESM.pdf]

Supplementary Information

Anti-obesity and lipid-lowering effects of Mountain Caviar Extract and its principal saponin momordin Ic via delayed gastric emptying and GLP-1 secretion independent of energy expenditure

Ryuya Takada[1] · Shogo Takeda[3] · Hiroshi Shimoda[3] · Motomitsu Tsukumo[4] ·

Kazutaka Kubota[4,5] · Hisashi Matsuda[1,5,6] · Toshio Morikawa[1,2]*

[1] Pharmaceutical Research and Technology Institute, Kindai University, 3-4-1 Kowakae, Higashi-osaka, Osaka 577-8502, Japan
[2] Antiaging Center, Kindai University, 3-4-1 Kowakae, Higashi-osaka, Osaka 577-8502, Japan
[3] Oryza Oil & Fat Chemical Co., Ltd., 1 Numata, Kitagata-cho, Ichinomiya, Aichi 493-8001, Japan
[4] ES Tech Kyoto, 15 Shimogamo Morimoto-cho, Sakyo-ku, Kyoto 606-0805, Japan
[5] Kyoto Organic Chemical Lab., 15 Shimogamo Morimoto-cho, Sakyo-ku, Kyoto 606-0805, Japan
[6] Research Institute for Production Development, 15 Shimogamo Morimoto-cho, Sakyo-ku, Kyoto 606-0805, Japan

* Correspondence: morikawa@kindai.ac.jp; Tel.: +81-6-4307-4306

Figure S1. Typical HPLC chromatograms of standard solution of momordin Ic (**1**, 500 μg/mL) and sample solution (2 mg/mL), and calibration curve of momordin Ic (**1**) ········ S1
Figure S2. ^{1}H-NMR spectrum of momordin Ic (**1**) in pyridine-d_5 ········ S2
Figure S3. ^{13}C NMR and DEPT135 spectra of **1** in pyridine-d_5 ········ S3
Table S1. Effects of MCE and momordin Ic (**1**) on pancreatic lipase activity ········ S4
Table S2. Effects of momordin Ic (**1**) on GIT in olive oil-loaded mice ········ S4
Table S3. Effects of administration of MCE and momordin Ic (**1**) on body weight, food intake, and TG content of feces in HFD fed mice ········ S5
Isotopic analysis in DLW measurement for energy expenditure ········ S6
Table S4. Effects of momordin Ic (**1**) on TEE using DLW method in HFD fed mice ········ S8

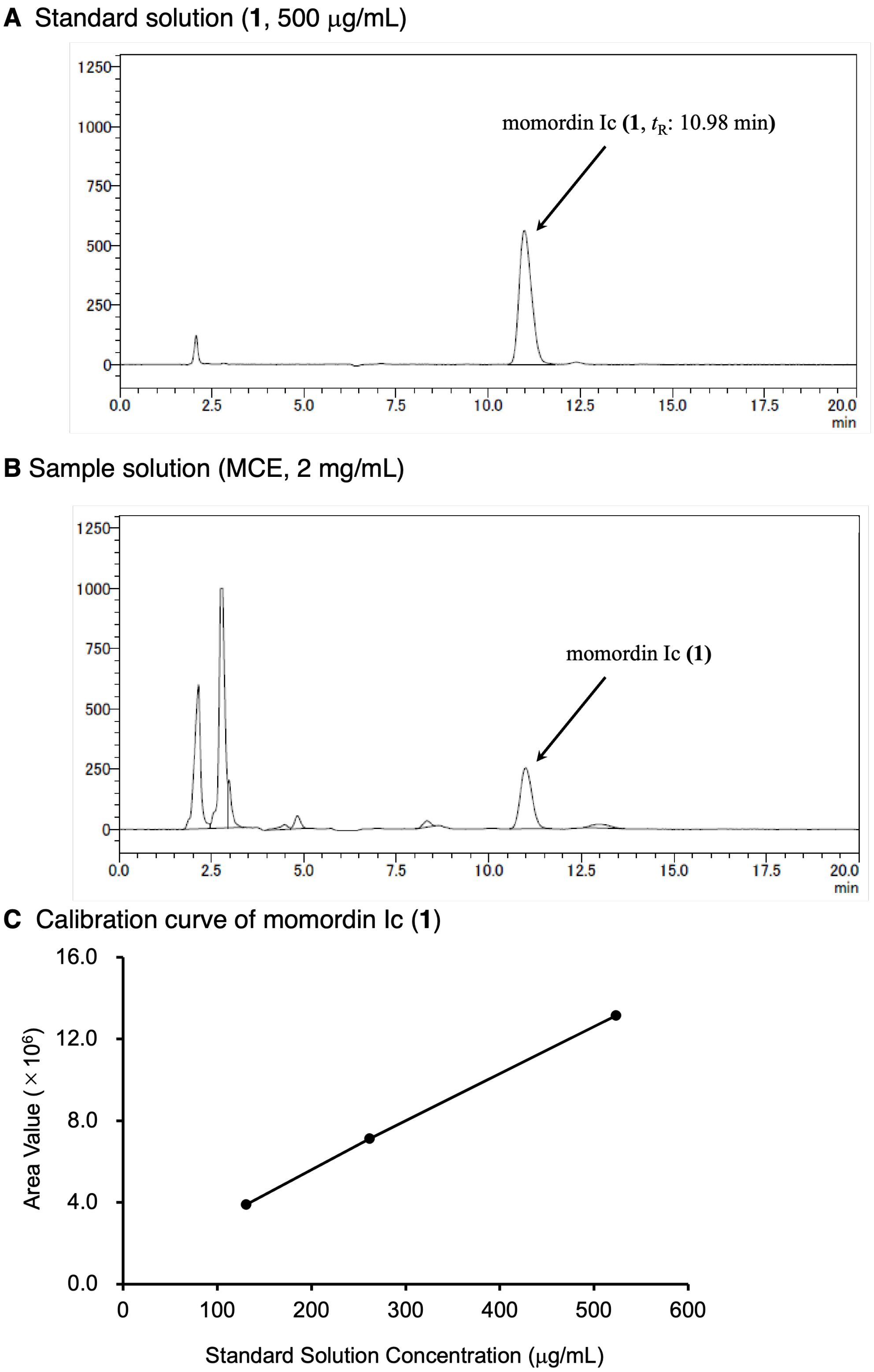

Figure S1. Typical HPLC chromatograms of standard solution of momordin Ic (**1**, 500 μg/mL) and sample solution (2 mg/mL), and calibration curve of momordin Ic (**1**)

momordin Ic (1)

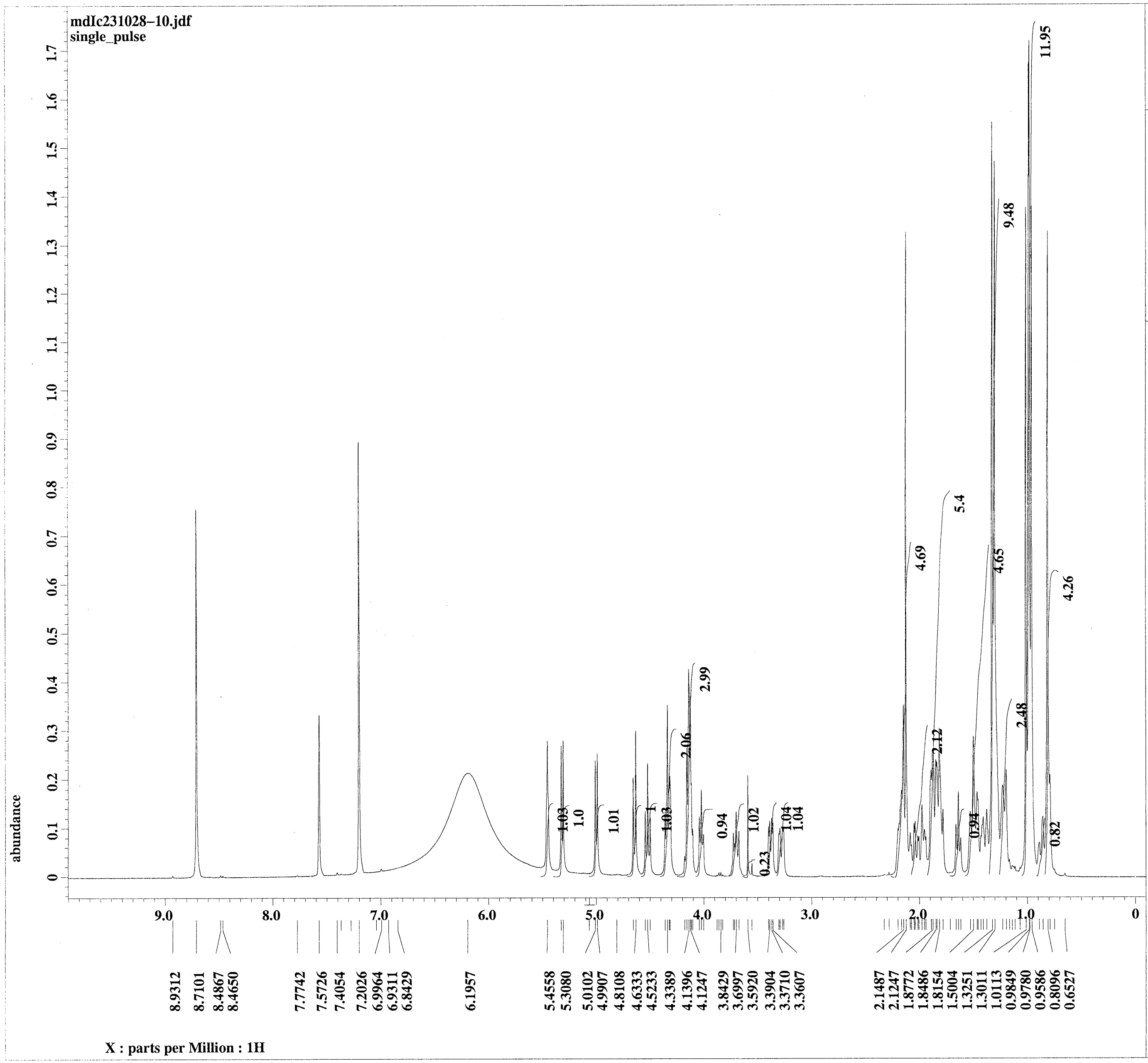

---- PROCESSING PARAMETERS ----
dc_balance : 0 : FALSE
sexp : 0.2[Hz] : 0.0[s]
trapezoid3 : 0[%] : 80[%] : 100[%]
zerofill : 1
fft : 1 : TRUE : TRUE
machinephase
ppm
reference : 8.646[ppm] : 8.71[ppm]

Derived from: mdIc231028-1.jdf

Filename	=	mdIc231028-10.jdf
Author	=	delta
Experiment	=	single_pulse.ex2
Sample_id	=	mdIc231028
Solvent	=	PYRIDINE-D5
Creation_time	=	6-NOV-2023 20:16:47
Revision_time	=	9-NOV-2023 18:58:42
Current_time	=	9-NOV-2023 18:59:18
Comment	=	single_pulse
Data_format	=	1D COMPLEX
Dim_size	=	13107
Dim_title	=	1H
Dim_units	=	[ppm]
Dimensions	=	X
Site	=	ECS 400
Spectrometer	=	JNM-ECS400
Field_strength	=	9.389766[T] (400[MHz]
X_acq_duration	=	2.18365952[s]
X_domain	=	1H
X_freq	=	399.78219838[MHz]
X_offset	=	5[ppm]
X_points	=	16384
X_prescans	=	1
X_resolution	=	0.45794685[Hz]
X_sweep	=	7.5030012[kHz]
Irr_domain	=	1H
Irr_freq	=	399.78219838[MHz]
Irr_offset	=	5[ppm]
Tri_domain	=	1H
Tri_freq	=	399.78219838[MHz]
Tri_offset	=	5[ppm]
Clipped	=	FALSE
Mod_return	=	1
Scans	=	32
Total_scans	=	32
X_90_width	=	11.7[us]
X_acq_time	=	2.18365952[s]
X_angle	=	45[deg]
X_atn	=	3.5[dB]
X_pulse	=	5.85[us]
Irr_mode	=	Off
Tri_mode	=	Off
Dante_presat	=	FALSE
Initial_wait	=	1[s]
Recvr_gain	=	36
Relaxation_delay	=	5[s]
Repetition_time	=	7.18365952[s]
Temp_get	=	40[dC]

momordin Ic (1)

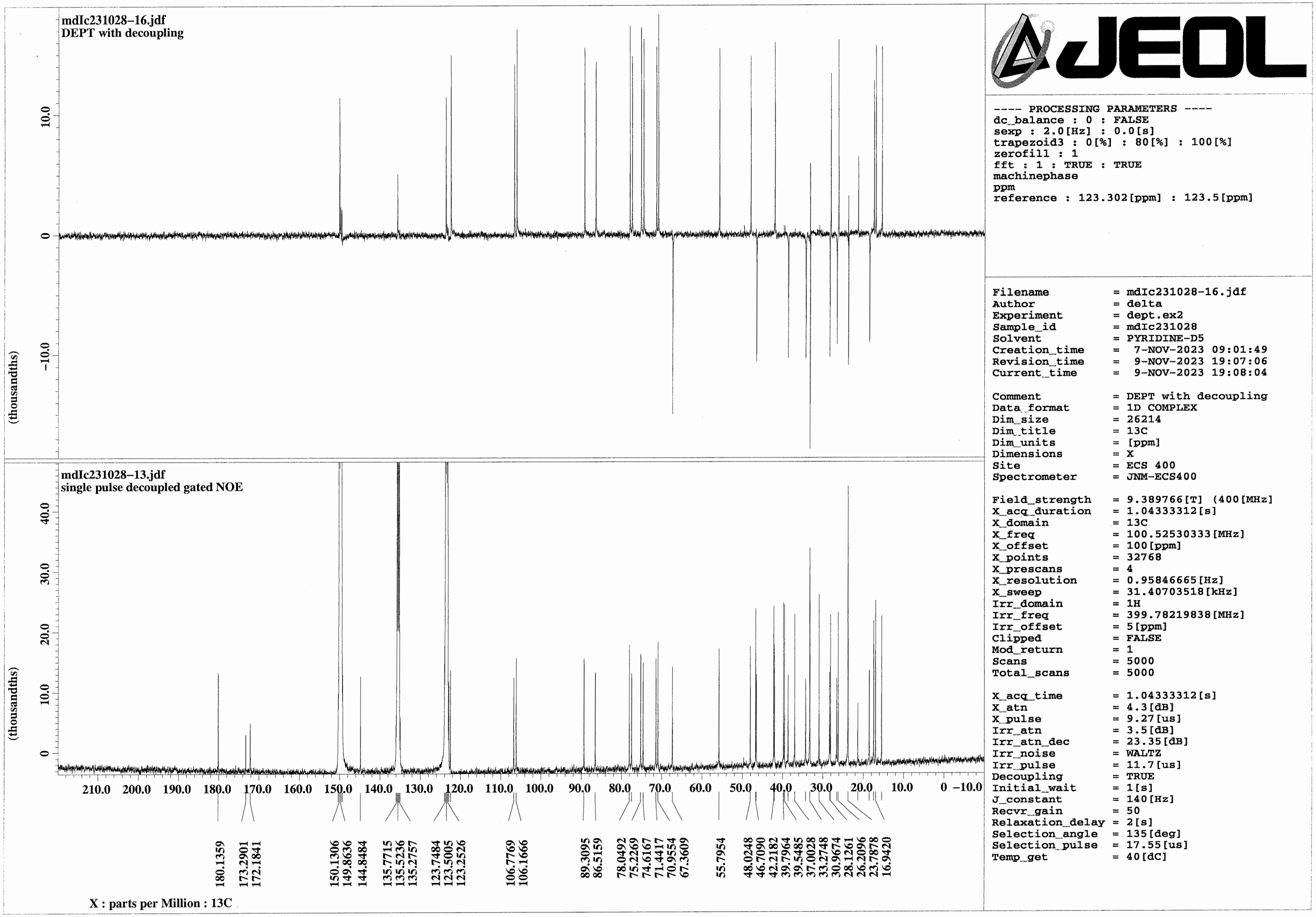

Table S1. Effects of momordin Ic (**1**) on pancreatic lipase activity

Conc. (μM)	Inhibition (%)					IC_{50} (μM)
	50	100	200	300	400	
Momordin Ic (**1**)	4.0	26.4	26.2	58.4	94.2	256.1

Conc. (nM)	Inhibition (%)					IC_{50} (nM)
	3	10	30	100	300	
Orlistat	40.0	61.3	83.5	89.5	94.9	5.1

Each value represents the mean of two experiments.

Table S2. Effects of momordin Ic (**1**) on GIT in olive oil-loaded mice

Time (min)	Gastraointestinal transit (%)			
	N	Control	*N*	Momordin Ic (**1**, 20 mg/kg, *p.o.*)
0	5	0.7 ± 0.7	4	2.3 ± 2.3
15	4	56.7 ± 6.1	7	32.0 ± 12.1
30	4	68.7 ± 5.0	5	65.5 ± 4.4
60	4	93.9 ± 3.9	4	83.1 ± 8.9
120	4	88.5 ± 11.5	5	91.1 ± 4.5

Significantly different from the control group, $^{*}p < 0.05$, $^{**}p < 0.01$.
Each value represents the mean ± S.E.M.

Table S3. Effects of MCE and momordin Ic (**1**) on body weight, food intake, and TG content of feces in HFD fed mice

Treatment	Dose (mg/kg/day, *p.o.*)	*N*	Average food intake[a)] (g/day)	(kcal/day)	Body weight (g) Day 1	Day 2	Day 3	Day 4	Day 5	Day 6
Normal	—	8	4.1	15.7	37.9 ± 0.3	40.7 ± 0.3*	41.2 ± 0.5	41.1 ± 0.5**	41.7 ± 0.6**	41.8 ± 0.5**
Contral	—	8	4.1	20.8	38.0 ± 0.2	42.3 ± 0.3	42.6 ± 0.4	43.5 ± 0.4	44.4 ± 0.4	45.1 ± 0.4
MCE	125	8	4.0	20.5	38.0 ± 0.4	41.2 ± 0.5	42.0 ± 0.6	42.0 ±0.6	42.0 ± 0.7*	42.6 ± 1.0*
	250	8	3.7	18.7	37.8 ± 0.3	40.8 ± 0.5	41.3 ± 0.5*	41.9 ± 0.5*	42.3 ± 0.5*	41.7 ± 0.8*
Normal	—	9	5.1	19.4	39.8 ± 0.5	43.6 ± 0.6	45.0 ± 0.6	45.9 ± 0.8	45.8 ± 0.8	46.5 ± 1.0
Control	—	9	4.4	22.1	39.5 ± 0.4	44.8 ± 0.5	45.5 ± 0.5	46.7 ± 0.6	47.9 ± 0.7	47.8 ± 0.6
Momordin Ic (**1**)	10	10	4.3	21.5	39.4 ± 0.4	44.4 ± 0.6	44.8 ± 0.6	46.1 ± 0.6	46.9 ± 0.6	47.1 ± 0.6
	20	10	4.6	23.1	39.6 ± 0.3	43.6 ± 0.5	44.7 ± 0.4	46.0 ± 0.4	46.7 ± 0.4	46.9 ± 0.6

Body weight (g) Day 7	Day 8	Day 9	Day 10	Day 11	Day 12	Day 13	Day 14	Day 15	Average of TG content of faces (g) 1st week (Days 3–5)	2nd week (Days 10-13)
41.7 ± 0.6**	41.8 ± 0.4**	42.1 ± 0.7**	42.4 ± 0.8**	42.6 ± 0.8**	42.3 ± 0.8**	41.8 ± 0.7**	42.4 ± 0.7**	39.4 ± 0.9**	3.5	2.5
45.6 ± 0.4	45.7 ± 0.4	46.2 ± 0.4	46.5 ± 0.5	46.8 ± 0.5	47.0 ± 0.6	46.7 ± 0.5	47.1 ± 0.6	44.9 ± 0.9	5.3	5.1
43.1 ± 1.1	43.3 ± 1.1	44.0 ± 1.2	44.4 ± 1.2	44.6 ± 1.3	44.7 ± 1.4	44.4 ± 1.5	44.7 ± 1.5	42.7 ± 1.6	5.2	4.6
42.1 ± 0.8**	42.1 ± 0.7**	42.4 ± 0.6**	43.0 ± 0.8*	43.2 ± 0.8*	43.5 ± 0.8*	43.6 ± 0.8	44.2 ± 0.9	42.4 ± 1.0	7.1	5.8
46.4 ± 1.1	46.8 ± 1.2	47.1 ± 1.3	46.9 ± 1.3*	47.0 ± 1.3*	46.7 ± 1.3	46.7 ± 1.3*	46.7 ± 1.3**	44.4 ± 1.4*	4.9	3.0
48.8 ± 0.7	49.3 ± 0.7	49.6 ± 0.6	50.0 ± 0.7	50.3 ± 0.7	50.8 ± 0.8	50.7 ± 0.9	50.7 ± 0.9	48.5 ± 0.7	7.9	4.8
48.3 ± 0.6	48.5 ± 0.6	48.3 ± 0.6	48.6 ± 0.5	49.2 ± 0.5	49.2 ± 0.5	49.3 ± 0.5	49.3 ± 0.5	47.2 ± 0.5	8.4	6.0
48.0 ± 0.5	48.0 ± 0.5	48.5 ± 0.7	48.6 ± 0.6	48.9 ± 0.6	49.7 ± 0.6	49.9 ± 0.6	50.2 ± 0.6	47.7 ± 0.6	8.4	8.6

[a)]Each value was calculated based on the food intake (g) measured from the two groups housed in two cages.
Male ddY mice were fed a high-fat diet (HFD-60) or normal diet (AIN-93M) for 14 days.
Each value represents the mean ± S.E.M.
Significantly different from the control group, $^{*}p < 0.05$, $^{**}p < 0.01$.

Isotopic analysis in DLW measurement for energy expenditure

Isotope analysis.

Plasma water was converted and analyzed for ^2H and ^{18}O atom% by isotope-ratio mass spectrometry (IRMS). The isotopic compositions of the diluted plasma (10-folds) were measured and converted to the undiluted value using

$$A = D(C - B) + B$$

where A is the undiluted concentration, B the diluent water, C the diluted measurement, and D the dilution factor.

Enrichment.

Isotopic enrichment at time t was calculated as the difference from baseline:

$$E_t = a_t - a_{\mathrm{pre}}$$

where a is atom fraction (atm%/100).

Elimination rate constants.

For each isotope, $\ln(E_t)$ was regressed against time t (days) using data at 4, 24, and 72 h according to

$$\ln(E_t) = \ln(E_0) - kt$$

and the elimination rate constant k (day^{-1}) was obtained as the negative slope (hourly values were converted by $k_{\mathrm{day}} = 24\,k_{\mathrm{hour}}$).

Dilution spaces and total body water (TBW, Q).

With dose water moles n_{dose}, dose atom fraction a_{dose}, baseline a_{pre}, and equilibrium atom fraction a_{eq} (4 h).

$$N = \frac{(a_{\mathrm{dose}} - a_{\mathrm{pre}})\, n_{\mathrm{dose}}}{(a_{\mathrm{eq}} - a_{\mathrm{pre}})}$$

This yielded N_d (^2H) and N_o (^{18}O). TBW was calculated with standard in-vivo exchange corrections:

$$TBW(\mathrm{mol}) = \frac{1}{2}\left(\frac{N_d}{1.041} + \frac{N_o}{1.007}\right)$$

The dilution spaces derived from deuterium (N_d) and oxygen-18 (N_o) slightly overestimate true total body water because of isotope exchange with non-aqueous hydrogen and oxygen in body constituents. Therefore, total body water was calculated using standard correction factors ($N_d/1.041$ and $N_o/1.007$) and expressed as the average of the two estimates.

CO_2 production.

Using the two-pool Speakman formulation [66,67],

$$rCO_2 = \frac{N_o k_o - N_d k_d}{2.078} - 0.0246\, N_d k_d$$

with k in day^{-1} and N in mol. Volumetric CO_2 production was
The divisor 2.078 represents an effective stoichiometric and fractionation correction factor accounting for the incorporation of two oxygen atoms into CO_2 and for isotopic fractionation associated with oxygen exchange between body water and CO_2, as described in the two-pool model of the doubly labeled water method. The term $0.0246\, N_d k_d$ represents a correction for isotopic fractionation associated with evaporative and respiratory water loss, which preferentially removes the lighter hydrogen isotope and causes an apparent overestimation of deuterium elimination. This correction factor was empirically derived and incorporated in the two-pool model of the doubly labeled water method.

$$VCO_2(\text{L/day}) = 22.4 \times rCO_2$$

(STPD approximation).

Total energy expenditure (TEE).
Respiratory quotient (*RQ*) was approximated by the food quotient (*FQ*) derived from diet composition (0.85 for standard chow, 0.75 for high-fat diet). Energy expenditure was computed by the Weir equation [68]:

$$TEE(\text{kcal/day}) = VCO_2\left(\frac{3.941}{RQ} + 1.106\right)$$

and normalized to body mass as kcal·kg^{-1}·day^{-1}.
The constants 3.941 and 1.106 in the Weir equation represent the average caloric equivalents of oxygen consumption and carbon dioxide production, respectively, derived from the stoichiometry of mixed substrate oxidation and accounting for protein metabolism.

Table S4. Effects of momordin Ic (**1**) on TEE using DLW method in HFD fed mice

Treatment	Dose (mg/kg/d)	N	Body weight (g)	TBW (mol)	k_d (×10^{-3}/h)	k_o (×10^{-3}/h)	rCO_2 (mol/d)	TEE (kcal/d)	TEE (kcal/kg/d)
Normal	—	6	35.5 ± 0.7	1.52 ± 0.02	9.0 ± 1.0	16.0 ± 1.5	0.120 ± 0.009	15.4 ± 1.2	435.3 ± 32.6
Control	—	7	37.0 ± 0.4	1.50 ± 0.02	10.1 ± 0.4	17.2 ± 0.4	0.118 ± 0.003	16.8 ± 0.4	454.6 ± 13.2
Momordin Ic (**1**)	10	5	35.2 ± 0.7	1.48 ± 0.04	9.3 ± 0.4	16.2 ± 0.5	0.113 ± 0.003	16.1 ± 0.4	458.9 ± 16.2
	20	4	36.3 ± 0.7	1.55 ± 0.07	10.0 ± 0.4	16.8 ± 0.4	0.115 ± 0.006	16.4 ± 0.8	450.4 ± 15.3

Each value represents the mean ± S.E.M.
TBW: total body water (mol), k_d: rate constant of ^2H (×10^{-3}/h), k_o: rate constant of ^{18}O (×10^{-3}/h), rCO_2: CO_2 production (mol/d)
Protocol: Male ddY mice (4-5weeks old) were fed a normal diet (AIN-93M) or high-fat diet (HFD-60) for 7 d.
The test sample was given orally once a day at 15:00–17:00.
DLW was administered 4 days before the end of the test.
The sample blood was collected at 0, 4, 24, and 72 h after administration of DLW.
